# From Online to Offline and Vice Versa: Change in Internet Use in Later Life Across Europe

**DOI:** 10.3389/fsoc.2020.00004

**Published:** 2020-02-18

**Authors:** Ronny König, Alexander Seifert

**Affiliations:** ^1^Institute of Sociology, University of Zurich, Zurich, Switzerland; ^2^Center for Gerontology, University of Zurich, Zurich, Switzerland

**Keywords:** ICT, internet usage, Europe, older adults, SHARE

## Abstract

The internet can be a valuable source of social participation in modern society. Although increasing numbers of older adults are using the internet, numerous older adults also cease using the internet and become “offliners.” The question of which factors in usage change (i.e., switching from onliner to offliner, and vice versa) are most influential remains unclear. This study investigates changes in internet use among the older European population using two waves of representative panel data for 13 countries from the Survey of Health, Aging and Retirement in Europe (SHARE). The analyses were based on 34,149 respondents aged 50 years and older. In general, the results indicate a positive trend by a slight (2%) increase in usage, from 51% in 2013 to 53% in 2015. However, the results also show that a total of 6% had not recently used the internet, although they had done so in the past. Our multilevel results suggest that both the onset of and cessation of internet usage are primarily driven by changes in socioeconomic resources (income and occupation), health resources (subjective health and grip strength), living situation (via relocation), and social factors (partnership and distance to children) as well as contextual factors such as country-specific wealth and social indexes and internet infrastructure. The study underlines the importance of investigating the influencing factors for commencing internet usage and of determining which factors lead to its increase.

## Introduction

Since the mid-twentieth century, modern societies have undergone a far-reaching process of transformation that has primarily been characterized by two developments. First, a demographic change has occurred (and will continue to occur) in which the proportion of the world's population over 60 will have doubled by 2050 (WHO, 2018). Second, a change has occurred in the form of the digitalization of everyday life, driven by high levels of technical innovation and diffusion dynamics through the use of information and communication technologies (ICT) such as the internet (Castells, 2010). Regarding the latter change, the current society is made up of networks as presented by the internet as a network of connected computers and therefore the internet dominates today societies (Castells, 2000) Nevertheless, specific segments of the population, mainly the older population, often do not have direct or immediate access to new technologies such as the internet and thus do not benefit as much from these technologies' advantages for daily living as those who do have such access (Hunsaker and Hargittai, 2018). This so-called digital divide may be understood as a globally applicable term for the perceived divide between those who have access to the latest information technologies and those who do not (Korupp and Szydlik, 2005). In recent years, the digital divide has shifted from the access/no access discussion toward a discussion about the second-level digital divide among people who have access to the internet (Büchi et al., 2016; Scheerder et al., 2017). Thus, scientific research has evolved to differentiate digital inequalities in internet use and therefore now focuses much more on questions regarding who is online, what onliners do with the internet in daily life, and which outcomes produce that internet use (van Dijk, 2006; van Deursen and Helsper, 2015). From this perspective, the question arises of who uses the internet and who does not. Along with socio-demographic characteristics (e.g., age, generation, gender, education, and income) and personal factors (e.g., health, attitudes toward technology, and anxiety about ICT), environmental circumstances such as ICT infrastructure also shape differences in technology use between the younger and the older population (Schulz et al., 2015; Hargittai and Dobransky, 2017; Berkowsky et al., 2018; Mitzner et al., 2019).

Although internet use via personal computers has become increasingly ubiquitous since the late 1990s—and more recently through the mobile internet, driven by the widespread diffusion of smartphones and tablets—individual internet use is still characterized by a digital divide among age and cohort groups (Hunsaker and Hargittai, 2018). Young age groups are currently embracing the internet, whereas adults who have not grown up using these technologies tend to use the internet less. Against this background and the fact that the internet is today the primary global form of ICT, the present study investigates the mechanism for changed internet behaviors (from online to offline and vice versa) among Europe's older population.

### Internet Use and Outcomes Among Older Adults

The internet has become the most important information and communication tool. Nevertheless, a digital divide between generations (meaning that older cohorts do use the internet less often than younger cohorts) remains, although several studies have noted an increasing trend of internet use among older adults (Hunsaker and Hargittai, 2018). For example, one recent study in the USA found that 67% of people in age of 65 years and older were online (Pew Research Center, 2017). While the divide in internet use between emerging and advanced economies has narrowed in recent years, some regions of the world—especially within developed countries—have significant numbers of older citizens, who do not use the internet (Pew Research Center, 2018). In contrast, a representative study of 17 European countries showed that 49% of people aged 50 and older used the internet (König et al., 2018). The findings indicated that internet use among older adults was influenced by socio-economic factors such as age, gender, education, and income. The study also highlighted that people over 80 years spent less time online than those in the slightly younger age group of 65–79 years and that men and older adults with higher educational and economical status were more likely to use the internet. In addition, people's health, prior experience with technology, social salience (i.e., internet use among the members of one's social network), and various contextual factors, such as country-specific wealth and ICT infrastructure have a lasting effect on internet use in advanced age (König et al., 2018).

Aging occurs in context, meaning that the environment of an older person can work as a barrier or a resource for maintaining everyday life and for aging in a healthy and successful manner (Lawton, 1983; Wahl and Lang, 2003). According to Wahl and Gerstorf (2018), technologies and digital resources such as the internet are important contexts for successful aging today. Internet use can have two outcomes in these cases. First, internet access can be viewed as a resource for coping with everyday life situations, which suggests that the internet could be a resource for promoting successful aging and for compensating functional declines in old age (Cotten et al., 2013; Forsman and Nordmyr, 2017; Sims et al., 2017). In this vein, internet usage seems to occur regardless of the limitations of old age, such as reduced mobility, sensory impairment, or living in a retirement home (Seifert et al., 2017). Second, lack of access to the internet (and thus to the information and services the internet provides) can result in the digital exclusion of older adults, especially in societies dominated by the internet and digital technologies in many areas of everyday life. This exclusion from participation in these areas of everyday life may also lead to subjective feelings of social exclusion (Seifert et al., 2018). Technology has the potential to perpetuate ageism: that is, older non-users of technology are typically viewed as frail or outsiders (Cutler, 2005), while older citizens who do not use the internet could become increasingly disadvantaged as the internet's societal pervasiveness progresses (Peacock and Künemund, 2007).

### Use Change: Becoming an Onliner or an Offliner

The digital divide in internet use and the potential outcomes and barriers of internet use in old age have been discussed intensively in both public and scientific research, although information about the determinants of constant or changed internet use in old age remains scarce (Hunsaker and Hargittai, 2018). The present study examines the question of why older adults become “onliners” (i.e., they have been using the internet recently and did not before) and “offliners” (i.e., they haven't been using the internet recently but did before).

Theoretical assumptions for which factors affect changes in internet use can be found at the micro, meso, and macro levels. First, from a sociological point of view, our micro level assumptions rely on the effect of social inequalities and having differential access to new technologies due to different socio-economic resources. Mingo and Bracciale (2018) have demonstrated, by means of the Matthew effect and in relation to the digital divide, a progressive digital impoverishment of the weakest sectors of the population, such as older people with lower socio-economic status. Although age is a primary contributor to the digital divide, previous research has highlighted that other factors such as income also affect internet use (Korupp and Szydlik, 2005; König et al., 2018). This scenario becomes even more crucial, when people (have to) leave the labor market, as unemployment benefits and pensions are usually lower than one's previous earnings from work. From a life-development perspective, older people are becoming more vulnerable, especially in the so-called fourth age (Laslett, 1994). Research has shown that one's health situation can hinder (when health status is low) or benefit (when health status is high) internet use (Choi and DiNitto, 2013; Kamin and Lang, 2018). Thus, not only age and socio-economic factors influence access to the internet, age-related factors such as health also affect the general accessibility in later life. Changes in one's socio-economic status or health situation can thus lead to a changed internet behavior.

Second, according to Lawton's (1983) eco-gerontological environment press model, the physical, economical, psychological, and social resources and competencies of older people are relevant determinants to be fit with environmental pressures. Older people with a lack of experience, competency, and social support with and accessibility to digital media can perceive the internet as a hindering environment with several barriers (Wahl and Gerstorf, 2018). Therefore, also social capital as captured from embedded resources in social networks (Lin, 2002) are important for adoption new technologies. In this vein, research has found that older adults who have social networks (e.g., relatives and acquaintances) and receive informal support and advice on internet use are more likely to use the internet on their own (Kamin et al., 2019) than those who lack these networks. Thus, having a social network of support (e.g., partners or children) can be seen as a resource for becoming online, whereas the loss of such relations and support can lead to internet-use stoppage.

Third, in addition to factors on the micro and meso level, macro level circumstances are also relevant for a person-environment fit. For example, relocations often require contacting one's current telecommunication provider in order to transfer an existing landline to the new place of residence; sometimes such moves even require a modification of the existing contract, with new services included. And, depending on the area of residence, spatial mobility often goes along with a changed network carrier for telecommunications such as TV and internet, the offers of which may include packages and services (such as complimentary internet access) with few or no costs. Relocations can thus lead people to become onliners and to benefit from advantages of the internet such as maintaining social contact with one's friends and neighbors from one's old place of living. In addition, because economically weaker and developing countries are often characterized by underdeveloped ICT infrastructures (Guillen and Suarez, 2005), a change in structural circumstances can lead to a change in individual opportunities and needs and thus affect the situation of becoming both an onliner or an offliner. Such factors may include country-specific economic development, the societal extent of poverty and inequality, and the expansion of national ICT infrastructure.

In sum, micro, meso, and macro level resources and circumstances can influence internet adoption among older adults. To our knowledge, no previous studies have focused on the predictors of changes in internet use, and more specifically on the chance of becoming an offliner. While most previous studies have explored internet usage—specifically the act of becoming an onliner—such use is merely one direction of internet use. The current study thus focuses on the micro, meso, and macro level predictors of older adults becoming recently both onliners and offliners.

### Research Question

In line with the theoretical review and current state of research as well as based on the concept of micro, meso, and macro level influences for using the internet among older adults as presented by a recent study of König et al. (2018), we have focused on the following main research question: “Which changes at the micro, meso, and macro level can explain changes in internet use in old age across Europe?”

According to the theoretical background and empirical findings of social inequalities and economic resources (see for example Mingo and Bracciale, 2018), our first hypothesis (**H1**) is that respondents who experience a positive change in their income situation will become onliners, while respondents who experience a decline in their income will become offliners. Because no employment such as unemployment or retirement is usually linked with a decline in income, we further hypothesize that respondents who have previously used the internet (for example at work) will stop using the internet once they retire or become not employed (**H2**).

Personal health is another important factor in old age. In accordance with eco-gerontologist concepts (Lawton, 1983), health is an important factor for reacting to environmental pressures. Older people with health issues, including visual, acoustic, and tactile limitations, are more likely to be offliners (König et al., 2018). We thus hypothesize that personal health status will be associated with individual internet use, meaning that a positive change of one's health status will promote internet use, while a decline of health will lead to the chance of becoming an offliner (**H3**).

For the influence of meso level changes, we hypothesize that changed social networks and/or conditions (i.e., the loss of a partner or the existence of spatial distance to one's children) will have a lasting effect on internet use. This assumption is based on the background of previous findings on social support for technology accessibility (Kamin et al., 2019). While the loss of a loved one (through separation or death) as one's primary social resource will not necessarily lead to internet-use stoppage, the sudden absence of one's partner's possible experiences and competencies with digital media may hinder the attaining of one's first internet experiences (**H4**). Although increased spatial distance to one's children can reduce accessibility to their digital knowledge, such distances do offer the chance (and need) to obtain the advantages of the internet to maintain family relations over distances which in turn results in a situation of start using the internet (**H5**).

Finally, on the macro level, we hypothesize that relocations and the associated contact with network providers and their offers will have a positive impact on becoming an onliner (**H6**). This not at least as new or modified contracts recently include a wide range of services (e.g., landline, television, internet, mobile services) in one package which in addition is mostly cheaper as one's previous contract. In addition, because people become more vulnerable with increasing age, we assume that a change of time—resulting in changes in life years—will lead to situations in which older cohorts are more likely to no longer use the internet (**H7**). For the contextual dimension, we also expect that changes at the societal level will also have an impact on internet usage in old age **(H8)**. In this vein, the existence of improved ICT infrastructure for promoting widespread internet access to the entire population will affect the older population's internet-usage behavior in a positive manner **(H8a)**. Simultaneously, an increase of inequality and poverty can lead to a situation in which a significant portion of the population—including those in old age—is no longer able to afford the costs associated with internet use **(H8b)**.

## Data and Methods

### Data

Our analyses are based on the Survey of Health, Aging and Retirement (SHARE), which provides standardized information on respondents aged 50 years and older in various European countries. The dataset employed in this study was gathered in the fifth (2013) and sixth (2015) waves (for details on the used data, see Börsch-Supan, 2019a,b) and contains information on a wide range of topics, including demography, income, health, accommodation, education, occupation, behavior, social support, activities, and expectations.

To capture the dynamics in internet use—namely, becoming an onliner or offliner over time—we used a two-wave panel design in which each respondent had participated in both SHARE waves. Our final sample consists of 34,149 respondents from thirteen European countries: Austria (*n* = 2,293), Belgium (*n* = 3,526), the Czech Republic (*n* = 3,270), Denmark (*n* = 2,943), Estonia (*n* = 3,290), France (*n* = 2,551), Germany (*n* = 3,515), Italy (*n* = 2,512), Luxembourg (*n* = 764), Slovenia (*n* = 1,438), Spain (*n* = 2,930), Sweden (*n* = 2,914), and Switzerland (*n* = 2,203).

The data structure must meet specific requirements to be able to transfer the situation of individual, familial, and contextual changes into a statistical model. A longitudinal design is necessary to cover interpersonal changes over time, while the construction of the dynamic behavior model must be based on a uniform definition (i.e., questioning) in each wave; variables that cover individual, familial, and contextual structures must also be available in sufficient cases across the entire period. These requirements were ensured by the existing two-wave panel design and were statistically embedded in a change score or first difference (FD) model. This method is based on the elimination of interpersonal similarities between two waves and focuses on the dynamics; this approach allows researchers to neutralize the transformation effect of the explanatory variables on the development process (Giesselmann and Windzio, 2012). To estimate changes for a person (within variance), each variable (dependent and independent) is subtracted by the chronologically previous eigenvalue. In line with the usually short observation period between two waves (in our case, 14–33 months, with a median of 23 months), the degree of change and therefore explanatory variance was restricted but could not be equated with any weakness in the model (Johnson, 2005; Allison, 2009).

Conversely, time-constant variables (e.g., gender and cohort) were included in their original form. Our interpretation of the effects is based on these variables' interactions with the factor of time. The advantage of this design is that, like all panel-analysis methods, it can expose the causal effects of an event on a dependent variable. Time-constant heterogeneities are also controlled for without the need for direct observation, and measurement errors are compensated by the model, both of which ultimately minimize estimation errors and distortions (Giesselmann and Windzio, 2012).

### Dependent Variable

The dependent variable was determined by respondents' internet use as derived from each interview, on the basis of the following question: “During the past 7 days, have you used the internet for emailing, searching for information, making purchases, or for any other purpose at least once?” The answer options were “yes” and “no.” The combination of the individual situation for each respondent and each interview allowed for identifying whether a change in their internet use—always in relation to the past 7 days—had occurred over time as well as the kind of change (if a change had indeed occurred). Unfortunately, the SHARE questionnaire only asked about internet use with this time-restricted binary item (yes/no). Therefore, our analyses can only include responses regarding the internet use based on the threshold of the past 7 days. Nevertheless, the time frame of 7 days represents an “active use” of the internet (Emmanouilides and Hammond, 2000), referring to a recent or even frequent user behavior. At this point, those respondents who had not used the internet at baseline but did during the follow-up were characterized as having become onliners, whereas the opposite situation of becoming an offliner was assigned if the respondent had used the internet at the time of the first interview but not at the second. The dependent variable was therefore based on the change score in internet use from both interviews and was differentiated according to direction: (a) no change, (b) becoming an onliner, and (c) becoming an offliner.

### Independent Variables

Various explanatory variables at the micro, meso, and macro level were included in the empirical models to explain such patterns in the European context against the background of change. Despite the short examination period, the possibility of the existence of temporary and opposing dynamics was taken into account. Time-variant variables were measured as the difference between the eigenvalues at both times of measurement, whereas categorical variables were dichotomized according to the state of change. A change in personal and economic needs was captured for the respondents' individual situations at the micro level. For the economic situation of respondents' households, a subjective income measure was used based on a question about whether the household had enough money to make ends meet. The answers fell into four graduated categories, ranging from “with great difficulty” to “easily.” On the basis of the information from both interviews, our analysis determined whether an income change had occurred; in those cases where a change had occurred, the specific direction (better or worse) was captured. Data on the labor-force status covered situations in which respondents had changed (a) from not having employment to having (self)employment (full or part time), (b) to a situation of no employment (such as becoming unemployed, homemaker, permanently sick or disabled) or (c) retired over time.

Because physical and mental health play important roles in the probability that one will or will not make use of the internet (König et al., 2018), we gathered this information using two indicators. First, we used a subjective self-evaluation of the person's actual health situation, measured on a 5-point scale (1 = *excellent*; 2 = *very good*; 3 = *good*; 4 = *fair*; 5 = *poo*r) for each wave. Second, to capture objective health conditions, we then gathered the respondents' maximum handgrip strength with the aid of a dynamometer. Both indicators allowed us to identify whether a change in individual health had occurred as well as the direction (better or worse) of that change over time. For the continuous measuring of grip strength, we defined a substantial change of objective health as having occurred if the respective value in the follow-up was differentiated by at least 10% from the initial value measured at baseline.

To capture meso level changes, the models included situations in which respondents had either entered into a new partnership or had lost a loved one—mainly through death—over time. As intergenerational contacts are strongly affected by geographical distance (Szydlik, 2016) and their frequency significantly increases with decreasing distance or decreases with increasing distance (König, 2016), we included the relevance of family spatial mobility as a further indicator for a possible change in internet use. To do so, we considered the importance of spatial mobility (and thus distance) as one key precondition for personal contact for respondents who had one or more children. In this vein, we captured the residential distance to the farthest-living child and each wave by the two distances of (a) up to or (b) above 100 km. While the first distance allows for more regular and personal contacts to be maintained, parent-child relations characterized by distances above 100 kilometers may rely more often on non-personal contact and thus on the communication opportunities based on the internet. Based on both indicators, a change in residential distance was recorded if the distances had become closer or farther over time. Responding parents without any change in residential distance between baseline and follow-up to their child or children, as well as those who were childless, were marked with the reference category “no change.” As previous findings indicate an increase in intergenerational contacts due to the birth of grandchildren (König, 2016), which in turn may affect the internet use of older adults, especially if their relatives live farther away, or a decline caused by spending more quality time with the newborn if it lives nearby, we included this indicator, capturing meso level changes on (not) using the internet recently.

Because a change in geographical distances within parent-child relationships might not result only from a child relocating, we further controlled for situations in which the respondents moved in terms of a macro-structural change within regions and thus countries. Moreover, we also considered the potential of time at the macro level to affect changes in individual behavior and included the months between both interviews as a continuous variable.

Although changes in structural conditions at the macro level such as ICT infrastructure, poverty, and inequality may show effects on individual internet usage being delayed, several indicators were considered in our analyses that captured variations in structural differences and societal inequality between European countries. In addition to the gross domestic product (GDP) per capita, adjusted for purchasing power, we used the level of internet access among the entire population (as a percentage of all households). We further included the poverty rate and the Gini coefficient to capture variations in societal inequality between countries. The poverty rate is defined as the ratio of the population whose income falls below 60% of the national median–equalized disposable income of the total population, whereas the Gini index measures the extent to which the distribution of income among households within a country deviates from an equal distribution. The macro indicators—drawn from official sources (e.g., Eurostat and OECD)—refer to the year preceding each interview for both waves (2012, respectively, 2014) and were entered into the models as the their change score as well as percentage change in order to consider the national levels at baseline.

Finally, a set of constant terms was controlled for in our analyses, including respondents' year of birth, gender (0 = female, 1 = male), and highest educational degree. Although the latter variable might change over time, we have assumed that education (as a proxy for cultural, economic, and social capital) will not change individual behavior over such a short period and especially not for respondents in age of at least 50 years (at baseline). The level of education was recorded according to the International Standard Classification of Education (ISCED) as *low* (ISCED 0–2), *medium* (ISCED 3–4), and *high* (ISCED 5 and higher).

### Methods

In addition to providing a descriptive overview of internet use in old age, we have also used multivariate analyses to explain which related factors account for such use. Given non-independence between observations (for example, because the respondents were nested in different countries), this hierarchical data structure would violate basic regression assumptions and might lead to inaccurate significance values as well as biased standard errors. To analyze the influence of changes in the determinants at the micro, meso, and macro level on becoming onliners or offliners, we estimated multilevel multinomial logit models (Hox, 2002; Rabe-Hesketh and Skrondal, 2012; Snijders and Bosker, 2012) involving two levels. In this way, we were able to account for variations at the upper country level. In addition to using the fixed or released parameters at the micro and meso level, these analyses included the context variables separately to avoid those estimation biases that stem from having multiple macro level indicators (Maas and Hox, 2005). Finally, all non-dichotomous variables were standardized and thus had a mean value of 0 and a standard deviation of 1.

## Results

### Trends in Internet Use in Old Age Across Europe: A Descriptive Overview

**Figure 1:**
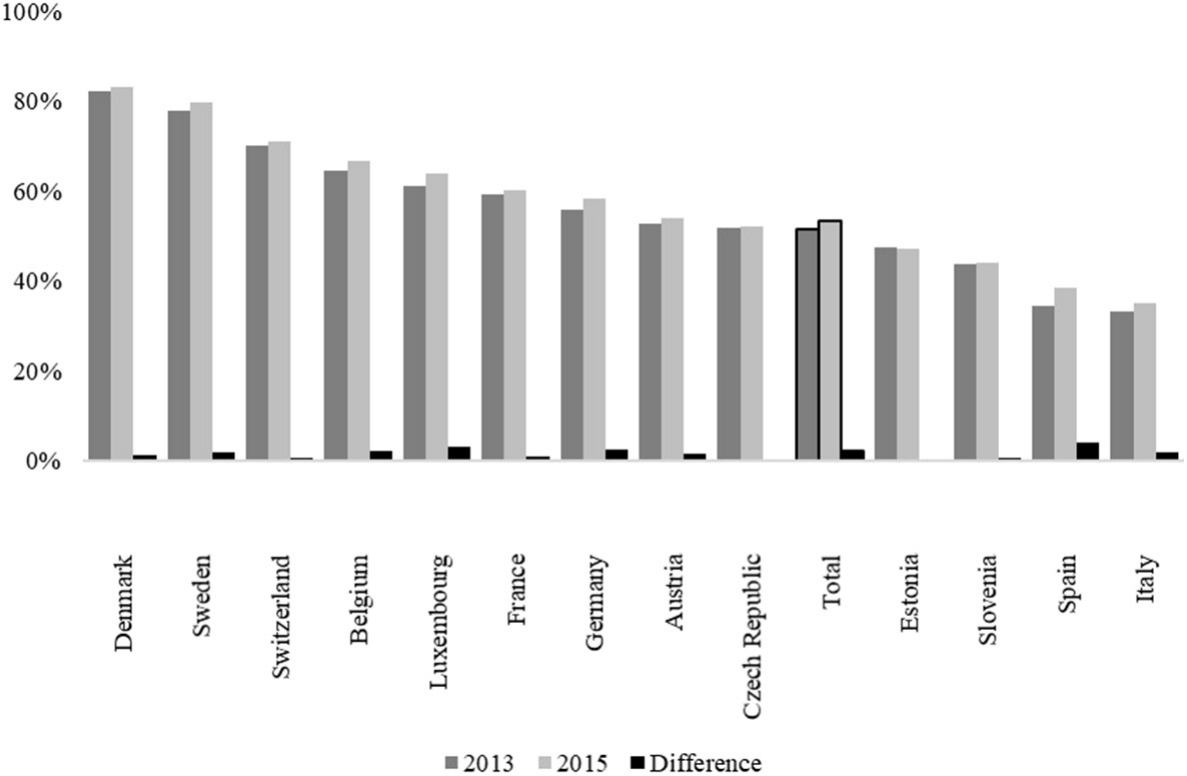
Trends in Internet Use in Old Age across Europe (Proportions). Footnote: Presented are proportions. Authors' own graph. Data source: Survey of Health, Aging and Retirement in Europe (SHARE), waves 5 and 6, release 7.0.0; *N* = 34,149; authors' own calculations, weighted. Countries sorted in descending order by internet use in 2013.

An initial glance at the distribution of internet use among older Europeans (Figure 1) shows that, overall, at least one of two respondents aged 50 and older were online in waves 5 (2013) and 6 (2015). For changes over time, the results further indicate a positive trend by a slight increase of two percentage points from 51% in 2013 to 53% in 2015 across Europe. Moreover, the results also show that a total of 6% stopped using the internet over that time meaning that those respondents were online during the past 7 days before survey conduction at baseline but not in the follow-up. While internet use in none of the 13 countries included in the study decreased significantly over that time, the increase was highest in Italy (+4%) and Luxembourg (+3%). In contrast, internet use remained at the same level in particular among Eastern European countries such as the Czech Republic, Estonia, and Slovenia between 2013 and 2015.

Although changes in internet use appear to be positive but low across Europe as well as within countries, the results also highlight a general and persistent north-south digital divide. While at least 80% of respondents in Denmark and Sweden had used the internet within the last 7 days in 2015, the extent of such onliners appears to have been lowest especially in Southern Europe (Italy and Spain), both with a proportion between 35 and 38%. The situation was almost similar for Eastern Europe (Estonia and Slovenia), where only 44–47% of respondents aged 50 and older used the internet in both 2013 and 2015. This is more astonishing as the respondents of the SHARE and thus of our sample are of a similar cohorts and age, which suggests that structural differences between these countries are a possible explanation for the different usage patterns across Europe.

**Figure 2:**
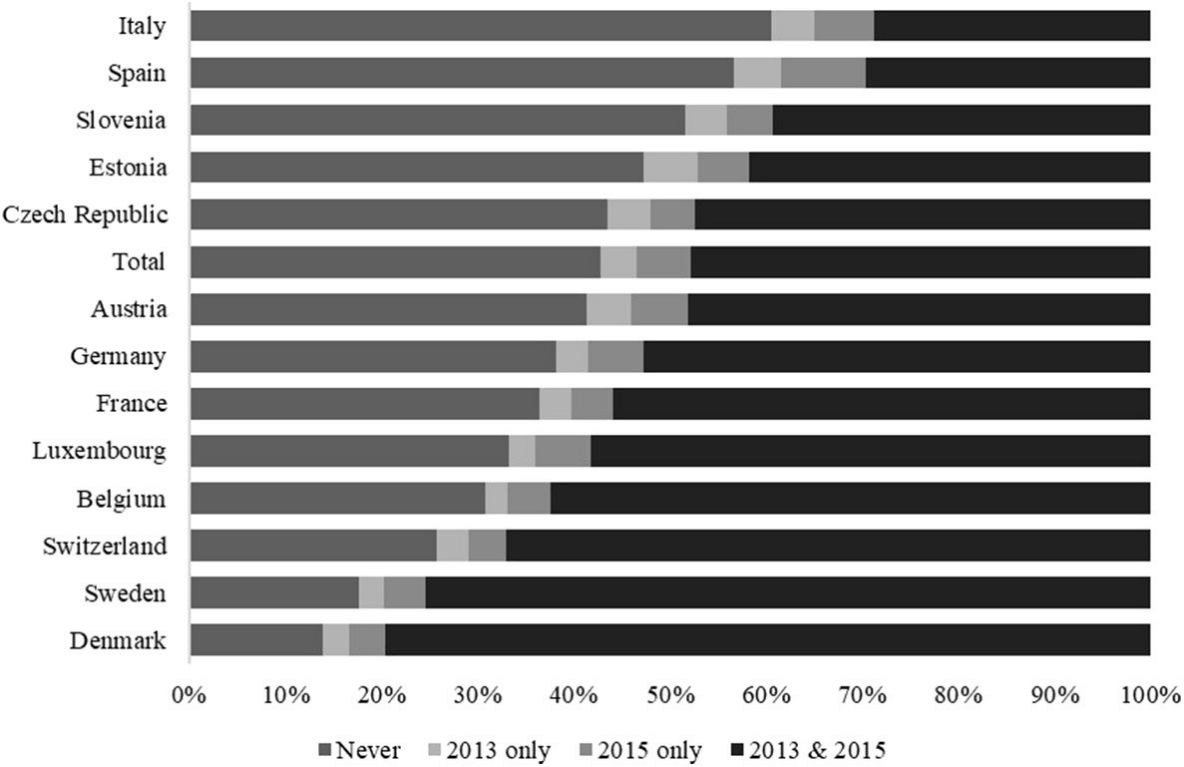
Internet Usage in Old Age over Time across Europe (Proportions). Footnote: Presented are proportions. Authors' own graph. Data source: Survey of Health, Aging and Retirement in Europe (SHARE), waves 5 and 6, release 7.0.0; *N* = 34,149; authors' own calculations, weighted. Countries sorted in descending order by internet use.

The number of onliners of advanced age seems to have been constant or even to have increased over time, although further analysis (see Figure 2) has highlighted the importance of individual changes in internet use. For example, the results show that 6% of respondents in Estonia, but also 5% in Italy, Spain, and Austria, didn't used the internet within the past 7 days anymore and thus stopped using the internet between 2013 and 2015, while 9% in Spain and 6% in Luxembourg, Germany, Austria, and Italy recently went online. Country-specific differences seem to have been more crucial, however, when we compared those who had never used the internet with those who reported to have been online in both waves. While only 14% of respondents in Denmark and Sweden had never used the internet recently, the extent of offliners appears to have been highest in Slovenia (52%), Spain (57%), and Italy (61%). We noted the exact opposite order when we focused on respondents who had used the internet in both 2013 and 2015: almost 80% of respondents in Denmark, followed by 75% in Sweden, reported such behavior, while the same was true for fewer than 30% in Spain and Italy.

### Determinants of Changes in Internet Use

The previous results indicate that a considerable proportion of older Europeans either start using or stop using the internet over time; the findings also suggest certain differences according to national context, however. To test our hypotheses and to explain which factors cause changed behaviors in recent internet use, we estimated multilevel multinomial logistic regression models that included indicators at the micro, meso, and macro level (Table 1).

**Table 1:** Determinants of changes in internet usage over time.

	**Became onliners**		**Became offliners**	
**Base:**	**No change**	**Became offliner**	**No change**	**Became onliner**
**Micro level changes**				
Income *(no change)*				
Better	1.11	1.19^**^	0.99	0.93
Worse	1.11	0.91	1.28^***^	1.16
Occupational situation *(no change)*			
Become employed	1.14	0.91	1.32	1.17
Become not employed	0.98	0.64^**^	1.55^***^	1.63^**^
Become retired	1.15	0.76^***^	1.51^***^	1.31^**^
Health (no change)				
Better	1.11^**^	1.13	0.99	0.92
Worse	1.04	0.90	1.16^***^	1.12
Grip strength *(no change)*				
Better	1.21^***^	1.24	0.99	0.82
Worse	1.12^*^	0.89	1.26^*^	1.13
**Meso level changes**				
Partnership *(no change)*				
New	0.83	1.06	0.78	0.93
Ended	0.70	0.70	0.99	1.38
Distance to child(ren) *(no change)*				
Closer	0.70^**^	0.54^**^	1.33	1.92^**^
Farther	1.25^***^	1.28	1.01	0.83
Becoming grandparent (again)	1.12^**^	0.96	1.17^**^	1.03
**Macro level changes**				
Relocation	1.13^*^	1.02	1.06	0.92
Months between T1 & T2	1.01	0.99	1.08^***^	1.10^**^
Δ GDP	0.94^***^	1.03	0.96	1.01
Δ Internet access	1.13^***^	0.95	1.20^***^	0.98
Δ Poverty	1.11^***^	0.99	1.18^***^	1.05
Δ Gini	1.06^**^	0.93^**^	1.11^*^	1.07
% GDP	0.95^**^	0.91^***^	1.05	1.14^*^
% Internet access	1.16^***^	0.96	1.24^***^	1.01
% Poverty	1.12^***^	0.99	1.18^***^	1.03
% Gini	1.01	0.92^**^	1.05	1.07^*^
**Constant terms**				
Male	0.90	0.80^***^	1.11^*^	1.24^***^
Year of birth	1.24^***^	1.07^*^	1.15^*^	0.94^*^
Education *(low)*				
Medium	1.05	0.87	1.11	0.96
High	0.59^***^	0.67^*^	0.73^*^	1.11
*N* (respondents)	34,149			
*N* (countries)	13			

*Data sources: Survey of Health, Aging and Retirement in Europe (SHARE), waves 5 and 6, release 7.0.0; Eurostat (2019a,b,c); OECD (2019); multilevel multinomial logistic regressions (separately for each macro indicator) and relative risk ratios (coefficients refer to the Δ GDP as macro indicator); all metric variables standardized; authors' own calculations. Significance levels*:

*** *≤ 0.01*,

** *≤ 0.05*,

* *≤ 0.10*.

In line with H1, a change in economic resources had a significant effect on internet use in old age. People in better financial positions are more likely to become onliners. Simultaneously, financial restrictions lead to changes in internet use: those respondents who reported a worse income situation between both interviews were more likely to become offliners as they didn't use the Internet (within the past 7 days) as before. This situation can be seen as a result of a change in respondents' occupational situation and highlights the linkage between employment and income. Following H2, our results confirm that entering in no employment such as retirement, unemployment, homemaker affects individual internet use in old age: specifically, new non-employed persons are both less likely to start and more likely to stop using the internet.

Similarly to financial resources, health plays an important role in internet use in old age (H3). Here, respondents who felt better but also showed significant improvements in objective health—measured by grip strength—became onliners over time, while the act of becoming an offliner was significantly affected by worsening health conditions. Increased physical health limitations, as well as the respondents' poorer subjective evaluations, together indicated that they had not been online recently, although they had used it before.

Along with micro level changes, variations at the meso level also affected internet behavior among older age groups, although changes within partnerships caused by either the loss of a partner or through entering a new relationship did not significantly change individual internet usage. Therefore, H4 cannot be confirmed. In comparison, the geographical closeness or distance to respondents' offspring seems to have promoted or prevented the usage of modern technologies such as the internet in later life, thus supporting the assumptions of H5. The multivariate findings show that respondents with decreased geographical distance to their children were both less likely to become onliners and more likely to become offliners over time. Simultaneously, an increase in geographic distance is associated with a high probability that the internet has been used recently. The importance of geographic distance to respondents' children for their internet behavior appears to be also important indirectly through grandparenthood. In this case, and probably depending on the distance, the birth of a (further) grandchild can lead to a diminution or cessation of internet usage in order to spend more time with the newborn or has been recently used to cover greater distances as a way of staying in contact.

Although the questionnaire did not allow for detecting if a change in geographical distance had resulted from a relocation of the responding parent, their offspring, or both, a further variable included information on whether the respondent had moved between both interviews. While relocations—as a kind of macro level change—do not necessarily lead to different residential distances, such moves can potentially change ICT usage. In line with H6, our results show that relocations can potentially cause people recently went online. In terms of time (and thus increasing age), our multivariate analyses confirm that the longer the time period between both interviews, the more likely the internet would no longer be used, thus supporting H7.

Previous research has emphasized the importance of structural conditions for being online in later life, such as better economic performance as well as generally higher internet access and thus usage among the entire population (König et al., 2018). Following these findings, our results have shown that a change in contextual circumstances can affect individual internet behavior and thus the chance of becoming onliners or offliners (H8a and H8b). In such cases, an increase in country-specific wealth (measured by GDP per capita) leads to situations in which older adults more often become offliners instead of becoming onliners. This situation applies especially to Estonia and the Czech Republic, both of which showed the largest economic development (+8%) for the observed period but at the same time had the lowest level of economic power in the European country comparison (Eurostat, 2019a). A similar picture and partially against our expectations (H8a) emerged when we focused on national expansions in the distribution of internet access. An increase in internet access in private households was found to have promoted changes in becoming onliners but also in becoming offliners. Although this finding may initially be surprising, it applies in particular to those countries that still have by far the lowest internet usage rates in Europe and mainly show at the same time more often a changed in Internet behavior such as the Czech Republic, Estonia, Italy, and Spain.

When we examined unequal opportunities and welfare-state interventions, the results showed that insecure circumstances have a lasting effect on individual behavior: for example, an increase in societal poverty will lead to changes in both directions, meaning that a considerable portion of the older population will start to use the internet, but others will also have to stop their usage. This situation may have mostly affected respondents from Estonia and Spain, who faced the strongest increase in poverty by at least two percentage points between both interviews and thus belonged to countries with the greatest societal poverty, with 26 and 29% (respectively) in 2014 (Eurostat, 2019b). In addition, a change in internet use (becoming recent onliners and offliners) also occurred more often in terms of increasing income inequality. When we differentiated the direction of change, however, the results also highlighted that the likelihood of becoming an offliner was higher during times of increased unequal income distribution compared to those who became onliners, and vice versa.

Finally, when we focused on the constant terms covering the respondents' socio-demographic and socio-economic backgrounds, we determined that males—who in general are more often onliners (see for example König et al., 2018) than females—are more likely to become offliners over time, while women tend to be catching up and thus are more likely to start rather than stop internet usage, always in relation to the past 7 days of the interview. The results further reveal that younger cohorts in particular were affected by changes in internet behavior: they were more likely to become onliners over time, but simultaneously they more often stopped internet use in private life. In addition to the gender factor (specifically, of being female), a lower social-class position appears to catch up over time in internet usage: respondents with higher educational background were less likely to change their behavior of already being online.

## Discussion

This study of older European adults describes changes in internet-use status—becoming online and offline—and the predictors of such changes at the micro, meso, and macro levels. While respondents who didn't used the internet within the past 7 days prior the baseline but 7 days prior the follow-up interview were defined as becoming onliners, the opposite behavior defines the situation of becoming offline. Overall, a major finding was the low but non-negligible percentages of older adults who not using the internet anymore. Those changes were influenced by alterations in personal resources, health-related and social circumstances, and contextual factors.

The results of the study's first and second hypotheses (H1 and H2), both of which address changes in socio-economic resources, show that economic resources have a significant effect on internet use. Specifically, positive changes in financial status were found to have led to a higher chance of beginning to use the internet, whereas a decline in this status was found to have led to a higher chance of remaining offline. This effect was also visible for the transition into retirement and no employment: those who had retired or became not employed were more often not recently using the internet than those who had not. These results are in line with previous findings, which have shown that financial resources are important for internet access among older adults (Korupp and Szydlik, 2005; Hong and Cho, 2017; Yoon et al., 2020).

H3, which focuses on possible changes in health status, was based on previous results that have shown that health can be a relevant factor in internet use (Choi and DiNitto, 2013; König et al., 2018). Our results have proved that health status plays an important role in internet usage during old age; more specifically, respondents who felt healthier and were healthier in terms of grip strength more often became onliners than those respondents who were less healthy. In turn, this situation means that those respondents who experienced declining health during the 2 years of the assessment were more likely to not using the internet recently. This scenario reflects the relevance of health-related resources for using the internet. Health factors play a role in determining who goes online and who stops being online. Especially among the oldest-old adults—who are typically confronted with more disabilities and activity-limiting symptoms and impairments than younger older adults—health-related limitations can result in stopping internet usage when that usage is based on the haptic use of computers or mobile devices such as smartphones. For example, vision impairment and memory limitations can be a crucial barrier for the use of many technologies (Gell et al., 2015). The internet might be a viable medium for health promotion when health-related information can be accessed online (Nutbeam, 2000), but older adults with certain types of impairment and disabilities seldom benefit from this positive outcome of internet usage because they are unable to use the internet without social support or assistive tools, such as voice control for computers for those with physiological limitations. When social support or assistive tools are non-existent, then the chance of internet usage is also limited.

When we then added the social component of internet usage, we assumed with H4 and H5 that a loss of a partner or a change in spatial distance to the respondents' child or children would affect internet usage. Although the results did not confirm H4 (loss of a partner), the related coefficients did confirm the expected direction, which suggests that taking a more differentiated view of the reasons for such changes will be necessary. This finding applies especially to terminated partnerships, which can result from the death of a loved one but may also occur through divorce or separation. The data used in this study, however, did not allow for distinguishing changed relationship statuses caused by separation or death for non-married respondents. In contrast, the findings have shown that geographical closeness or distance to respondents' offspring seems to promote or prevent internet use, thus supporting H5. The multivariate findings have shown that respondents with decreased geographical distance to their children were both less likely to become onliners and more likely to become offliners over time. Geographically closer relationships do not seem to rely on the internet's advantages for staying in contact, for example, and are likely more typical among personal interactions especially if grandchildren are born. In terms of increased residential distance within parent-child relationships, however, the results have shown that such circumstances tend to cause people to take advantage of the internet. As soon as greater distances make personal contact more difficult, the internet proves to be an adequate possibility for maintaining contact. Still, a discussion has arisen in the last decade about the promise of digital connections in comparison to personal social contact among older adults (Antonucci et al., 2017). Future studies thus should focus not only on the various types of (digital) social contact available today but also on the quality and feelings of attachment within those connections.

Finally, on the macro level, we assumed that changes in the contextual situation would have an effect on changes in internet-use status. In line with H6, our results show that relocations can potentially cause people to become onliners. Relocations often require contacting network providers to change billing addresses and to transfer landlines, and they often result in better offers and contracts. In terms of time (and thus increasing age), our multivariate analyses confirm H7, which posits that the longer the period between both interviews conducted in the study, the more likely it would be that respondents would no longer be using the internet.

For the country-related contextual dimension, we expected that structural changes would also have an impact on internet use (H8). Our findings point to the importance of country level stability and that comparatively strong fluctuations or uncertainties at the country level can potentially alter individual behavior in both directions (becoming onliners and offliners). In line with previous findings (König et al., 2018), however, societal prosperity, reduced inequality and poverty, and, in particular, a well-established ICT infrastructure can be seen as a general environment in which people use the internet not only once but over longer periods of time, even in old age. This finding falls within the scope of Lawton's (1983) eco-gerontological environment press model, that new environments, such as new ICT infrastructure can have a salience effect on older adults' adoption of technologies; such technologies included the internet and other new ICT devices such as smartphones or smartwatches (Wahl and Gerstorf, 2018).

Although we evaluate the change in internet use and argue that ICT usage can be a resource for maintaining everyday life in old age, we simultaneously do not argue that its non-use can be treated as an exclusion from digital social life. Moreover, the decision not to use the internet can be seen as individuals' conscious decision (Selwyn, 2003; Reisdorf and Groselj, 2017). In line with the actor-network theory, (Tatnall and Lepa, 2003) argued that the interactions with non-human entities like internet connections are not less important than interactions with other humans and that the adoption of technologies is not directly linked with any supposedly innate characteristics of technology but rather that unspecific uses of this technology relate to individuals' social interactions and environments. Therefore, it is not only important to investigate the change in internet use over time, but to also investigate how older adults negotiate with technologies such as the internet and how technology negotiates with them and how it affects older adults' own view of their lives and the experience of aging (Wanka and Gallistl, 2018).

### Limitations

As the present study focused specifically on Europe, the ability to generalize our findings outside Europe may be limited, and because the dependent variable was binary, not all possible facets of changes in the intensity of internet usage (e.g., different levels of use) could be taken into account. A limitation arises regarding the dependent variable used for our analyses: “During the past 7 days, have you used the internet for emailing, searching for information, making purchases, or for any other purpose at least once?” Unfortunately, the SHARE dataset only measured internet use with this question. Therefore, information about the stopping or starting of internet use are limited to the information about the past 7 days. Our analysis, therefore, could only distinguish between people who have reduced or increased their usage over the years. Nevertheless, we believe that using the SHARE dataset helps us to investigate the point of change in user behavior, even with some limitations. Hence, future studies are needed to measure internet behaviors and their change or stability over time in more detail.

Although the SHARE data allows us to investigate the internet use or non-use for a variety of European countries and especially among older adults, the survey is also lacking in several important variables, such as technology biographies, attitudes toward technology, technology acceptance, the use of technology in the household, and people's reasons for non-use. Future studies with representative data should thus investigate the influencing factors on changes in internet status more deeply through the use of longitudinal studies. Moreover, international data is needed to learn more about the digital divide, including whether the divide emerged between age cohorts and/or countries as well as their development over time. Furthermore, qualitative methods should also be used to study the daily internet use among older adults in more detail and should inquire into the reasons for use or non-use as, for example, presented by Mitzner et al. (2010).

Despite these limitations, increasing numbers of older people will undoubtedly use the internet in the near future. The digital divide will decrease in terms of both period effects (in terms of technological evolution and requirements) and cohort effects (in terms of educational expansion and increasing technical experience at work). The study's finding that older adults also stop using the internet should lead gerontology researchers in particular to be sensitive to the topic of social exclusion because of the phenomenon of digital exclusion. Future studies should investigate the reasons for stopping internet use and should also discuss possible interventions for addressing those older adults in particular.

Although the most recent seventh wave of the SHARE survey includes a question regarding internet use within the past 7 days, we excluded those interviews for several reasons. First, the survey conducted in 2017 primarily followed the “SHARELIFE” questionnaire and thus focused on people's life histories with retrospective questions. In this case, only those respondents who had already participated in the first SHARELIFE wave (2008/11) had to answer the main SHARE questionnaire, including the item of interest on internet behavior. Using this data would have drastically decreased the available number of participants by over 80% as well as compromising the comparability to the fifth and sixth waves. Second, using this information might have had considerable consequences, because those respondents who had already participated in one of the first SHARE waves were restricted to a specific number of countries as well as to older cohorts, which in itself could have led to sample-selection bias.

## Conclusion

For the European countries considered in this study, the findings provide support for the importance of investigating the influencing factors of changes in technology usage in old age in both directions: becoming a user and becoming a non-user of the internet. According to the results of the present study, changes in internet-use status are driven especially by socio-economic, social, and health-related resources. For the older population, interventions should promote internet use (such as skills training) and should focus on people's life courses, social networks, and country characteristics.

## Data Availability Statement

The datasets generated for this study are available on request to the corresponding author.

## Author Contributions

All authors listed have made a substantial, direct and intellectual contribution to the work, and approved it for publication.

### Conflict of Interest

The authors declare that the research was conducted in the absence of any commercial or financial relationships that could be construed as a potential conflict of interest.
